# The generation of novel epialleles in plants: the prospective behind re-shaping the epigenome

**DOI:** 10.3389/fpls.2025.1544744

**Published:** 2025-03-21

**Authors:** Alessio Baldini, Filippo Battaglia, Giorgio Perrella

**Affiliations:** Department of Biosciences, Università degli Studi di Milano, Milan, Italy

**Keywords:** gene editing, CRISPR/Cas9, zinc finger proteins, TALEs, DNA methylation, histone modifications, Arabidopsis

## Abstract

Chromatin organization is a relevant layer of control of gene expression during plant development. Chromatin states strictly depend on associated features such as DNA methylation, histone modifications and histone variants. Thus, epigenome editing has become of primary interest to alter gene expression without disrupting genomic sequences. Different tools have been developed to address this challenge, starting with modular Zinc Finger Proteins (ZFPs) and Transcription Activator Like Effectors (TALEs). However, the discovery of CRISPR/Cas9 system and the adaptability of technologies based on enzymatically dead Cas9 (dCas9) have paved the way towards a reliable and adaptable epigenome editing in a great variety of organisms. In this review, we will focus on the application of targeted epigenome editing technologies in plants, summarizing the most updated advances in this field. The promising results obtained by altering the expression state of targets involved in flowering time and abiotic stress resistance are crucial not only for elucidating the molecular interactions that underly chromatin dynamics, but also for future applications in breeding programs as an alternative route to genetic manipulation towards the achievement of higher quality crops particularly in terms of nutritional properties, yield and tolerance.

## 1 Introduction

In eukaryotes, DNA is associated in the nucleus with histone proteins to form a complex known as chromatin. Histone core octamer is formed by two tetramers consisting of histones H2A, H2B, H3 and H4, which allows to the DNA to be packed (Luger et al., 1997). This basic organization later develops in higher-order structures, from larger fibers to chromosome domains (Schubert et al., 2014). Epigenetic features such as DNA methylation, histone modifications and histone variants are therefore essential to compartmentalize and shape chromatin into distinct states (Kharchenko et al., 2010; Sequeira-Mendes et al., 2014). Their role in controlling the packaging of chromatin allows them to regulate the accessibility of DNA to facilitate the binding of transcription factors, ultimately leading to a finely tuned regulation of gene expression (Mansisidor and Risca, 2022). As a first layer of epigenetic control, DNA can be methylated. Methylation mainly occurs on cytosines, which results in 5-methylcytosine (5mC), a repressive mark highly employed by all eukaryotes. In plants, 5mC can be found in all contexts (CG, CHG and CHH, where H=A, T or C) (Schmitz et al., 2019). DNA methylation can be maintained or added *de novo* by the RNA-dependent DNA methylation (RdDM) pathway (for more information, see Zhang et al. (Zhang et al., 2018)). In this pathway, the RNA Polymerase IV (Pol IV) synthesizes single-stranded RNAs (ssRNAs) specific to the target locus (Onodera et al., 2005). These ssRNAs are then converted into double-stranded RNAs (dsRNAs) by RNA-DEPENDENT RNA POLYMERASE 2 (RDR2) (Haag et al., 2012). The resulting dsRNAs are processed by DICER-LIKE 3 (DCL3) into 24-nucleotide small interfering RNAs (siRNAs) (Henderson et al., 2006; Loffer et al., 2022). SiRNAs are then bound by ARGONAUTE proteins 4 and 6 (AGO4/6), forming a complex capable of recognizing non-coding RNAs (ncRNAs) synthesized by RNA Polymerase V (Pol V) at the target locus (Duan et al., 2015). The Pol V-ncRNAs-AGO4/6-siRNAs complex then recruits DOMAINS REARRANGED METHYLTRANSFERASE (DRM) 1 and 2, which catalyze methylation on defined loci (León-Ruiz et al., 2023). Additionally, factors like SAWADEE HOMEODOMAIN HOMOLOG 1 (SHH1), a histone reader, can recruit Pol IV to chromatin (Law et al., 2013), while DNA methylation readers SUVH2 and SUVH9 are responsible for recruiting Pol V (Johnson et al., 2014).

DNA methylation has been observed to crosstalk with histone modifications in feedback mechanisms (Cedar and Bergman, 2009). Histone modifications are typically deposited on the N-terminal tail of histones and are broadly responsible for fine regulation of gene expression. Among them, histone acetylation is deposited by histone acetyltransferases (HATs) and removed by histone deacetylases (HDACs) that can occur on the four core histones on lysine residues (Shen et al., 2015). While the function of some histone modifications might change depending on their chromatin context, others tend to have a specific role. For instance, histone acetylation is primarily related to gene expression (Lauria and Rossi, 2011). Additionally, histones can also be methylated. Indeed, methylation of lysine 27 of histone 3 (H3K27me3), works as repressive mark deposited by the multimeric Polycomb repressive complex 2 (PRC2), whose catalytic subunits deposit H3K27me3 through their Suv(ar) Enhancer of Zeste, Trithorax (SET) domain (Kim et al., 2012). The SET domain is shared by other histone methyltransferases such as KRYPTONITE/SUPPRESSOR OF VARIEGATION 3–9 HOMOLOG 4 (KYP/SUVH4). SUVH4 is also responsible for H3K9me2, typically associated with heterochromatin. Methyl groups are removed by the histone demethylases belonging to the JumonjiC (JmjC) family of Fe (II)-dependent and 2-oxoglutarate-dependent dioxygenases (Crevillén, 2020). While these modifications are among the best studied, a detailed description of the other modifications is given by Candela-Ferre et al. (Candela-Ferre et al., 2024).

**Figure 1:**
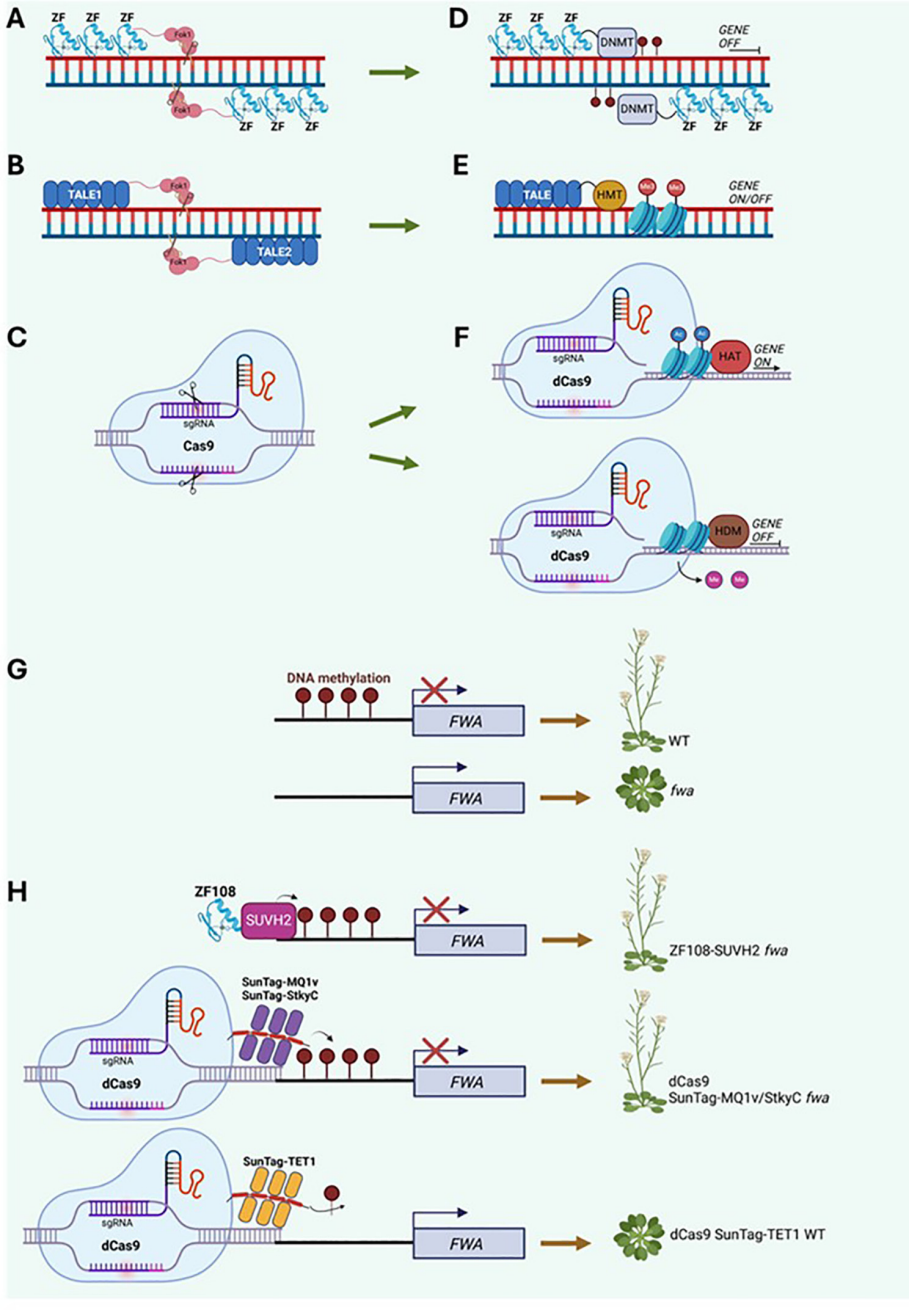
Genome and Epigenome editing strategies. **(A–C)** Depiction of the established gene editing tools in eukaryotes: **(A)** Zinc Finger Nuclease, comprising of three to six DNA binding domains fused to a Fok I endonuclease with cutting activity; **(B)** TALEN (Transcription Activator-Like Effector Nuclease), generated by fusing TALE effector DNA-binding domains to the endonuclease Fok I; **(C)** Clustered Regularly Interspaced Short Palindromic Repeats (CRISPR) Cas9 which targets DNA double strand breaks using a small guide RNA (sgRNA) located within RNA scaffold and Cas9 endonuclease that makes cuts in the appropriate region. **(D–F)** Epigenome editing can occur via a modified ZFN fused to a DNA methyltransferases (DNMT) deposited over target sequences **(D)**; TALE can be combined with histone methyltransferases (HMT) and promote or repress gene expression **(E)**; inactive (dead) dCas9 coupled with sgRNA can be combined to enzymes such as histone acetyltransferases (HAT) or histone demethylases (HDM) to prompt acetylation or remove methylation, hence gene regulation **(F)**. **(G)** Case study of the FWA locus in Arabidopsis, whose DNA methylation (m5C) state in WT determines flowering initiation. Conversely, fwa plants shows hypomethylation which causes late flowering. **(H)** DNA methylation can be restored by ZF fused to m5C reader SUVH2 in *fwa*; alternatively, *FWA* DNA epistate is modified via dCas9 with sgRNA associated with a single chain variable fragment (scFv) that is part of a repetitive protein scaffold known as SunTag. SunTag can recruit several copies of bacterium DNA methyltransferase MQ1v, m5C binding domain StkyC or human demethylase TET1, leading to *FWA* hyper or hypomethylation.

Recently, epigenetic features are emerging as primary targets to alter plant responses towards stresses, functioning as a means for crops to face adverse conditions. Multiple epigenomic editing tools have been developed so far, and while most of them are well established on animal cells, reports are now increasing about their employment in plants. These tools rely primarily on technologies first developed for targeted genetic modifications, later re-shaped to edit epigenetic features (**Figure 1**). Given its simple application, efficiency and plasticity, Clustered Regularly Interspaced Short Palindromic Repeats (CRISPR) and CRISPR-associated protein (Cas) quickly overcame previous epigenome editing tools, which mainly relied on modified nuclease systems. One of the first systems employed was based on Zinc Finger proteins (ZFPs), which belong to a family of transcription factors that contain linear repetitions of Cys2His2 (C2H2) zinc-finger DNA-binding domains (DBDs) (**Figure 1**). In coordinate a zinc ion for a total of 7 to 11 ions per ZFP (Miller et al., 1985). Each ZF contains 30 amino acids that form a conserved ββα structure, whose α-helix is responsible for recognizing a triplet of nucleotides (Beerli and Barbas, 2002). The binding of target sequences is mostly independent from other ZFs, although some exceptions have been reported (Segal et al., 2003). These features were therefore considered to design multi-ZF domains to bind sequences in a first attempt to target specific sites, starting with engineered Zinc Finger Nucleases (ZFNs), fused to nuclease domains such as FokI to introduce double strand breaks and trigger repair mechanisms (Guo et al., 2010). Later applications replaced nucleases with other catalytic domains, such as Ten-Eleven Translocation (TET) 5mC oxidases, involved in 5mC removal (Chen et al., 2014). These experiments suggested ZFs as a valid starting point for epigenome editing even though this system was observed to bind a large number of off-target loci on a genome-wide level (Grimmer et al., 2014).

A second approach for targeting specific sequences employs Transcription Activator-Like Effectors (TALEs) secreted by the plant bacteria *Xanthomonas oryzae*, which normally activates genes that promote bacterial growth (Boch and Bonas, 2010). TALEs DBDs consist of a variable number of tandem repeats, each binding to a specific nucleotide (Jankele and Svoboda, 2014). The fusion of tandem repeats therefore allows to generate synthetic TALEs with unique consensus sequences, serving as a scaffold for functional domains such as nucleases, transcriptional activators or repressors and epigenetic machineries (Hensel and Kumlehn, 2019). While the modularity of TALEs makes them an easier tool to engineer compared to ZFs, with commercial kits that also allow high-throughput TALEs synthesis (Cermak et al., 2011), the high number of tandem repeats that is required to clone for their functionality is a challenging obstacle that prevents their extensive use for targeted editing purposes.

Despite efficiently acting as epigenome editors (Siddique et al., 2013; Gao et al., 2014), TALEs and ZFs-based systems presented common drawbacks that prevented their extensive use. Most of all, the relative complexity of synthesizing factors for specific sites does not allow targeting multiple loci at once, reducing the multiplexing capabilities of both ZFs and TALEs (Cano-Rodriguez and Rots, 2016).

A huge boost in the editing field was prompted by the discovery of the CRISPR/Cas system, which, eventually replaced the previous tools based on engineered proteins. The CRISPR system is an adaptive defense mechanism naturally found in prokaryotes that protects the cell from bacteriophages and plasmid DNA (Garneau et al., 2010). This defense mechanism is based on the presence of a locus CRISPR, consisting of repeats separated by spacers. These spacers derive from exogenous genetic material that the prokaryotic cells can recognize and integrate in the CRISPR locus to activate a targeted degradation upon a second exposure (Barrangou et al., 2007). *Streptococcus pyogenes* is the model organism where the CRISPR system has been widely studied. In this bacterium, a type II CRISPR mechanism is present, requiring two different RNAs to properly work: crispr RNA (crRNA), encoded by spacers, and a small trans-activating crispr RNA (tracrRNA), essential for the maturation of pre-crRNA to mature crRNA. Pre-crRNA binds to nuclease Cas9 and matures into crRNA through the activity of tracrRNA-directed RNAse III, leading the complex towards exogenous genetic material to degrade by recognizing specific consensus sequences known as Protospacer Adjacent Motifs (PAMs) (Charpentier et al., 2015). Later studies led to the transfer of this system to other prokaryotes to provide defense towards exogenous nucleic acids (Sapranauskas et al., 2011). The promising results obtained therefore opened to the possibility of employing CRISPR/Cas as a tool to target endogenous genomic material in the host cell by designing proper guide (g)RNAs. Indeed, the breaks on the DNA caused by the Cas9 activate the repair machinery in the cell, thereby introducing mutations. In this context, experiments led on bacteria and human cells demonstrated its versatility and its efficiency, even by targeting multiple loci at the same time via multiple gRNAs (Jiang et al., 2013; Mali et al., 2013). Furthermore, the introduction of targeted double strand breaks in the host DNA can also lead to repair errors or homologous recombination to trigger gene knock-out and homology-based insertions (Hsu et al., 2014), supporting the role of CRISPR/Cas9 as an efficient golden standard system for genome editing in all organisms. Later refinement of this tool resulted in its further enhancement, introducing the possibility of using single guide RNA (sgRNA) and targeting an increasing variety of PAMs (Swarts and Jinek, 2018) Based on this premise, CRISPR/Cas9 system has been repurposed to expand its function, due on its unprecedented versatility for other forms of targeted editing.

Dead Cas9 is a Cas9 deprived of its DNA cleavage domain developed by Qi et al. (Qi et al., 2013). It was first used as a tool to repress gene expression by targeting gene promoters to hinder RNA polymerase occupancy and reduce its activity, ultimately leading to expression interference without altering genomic sequences. Later applications of this tool employed dCas9 fused to different catalytic domains including the endonuclease FokI for more efficient genetic manipulation (Saifaldeen et al., 2020) but also combined with DNA demethylases for the removal of DNA methyl groups (Wang et al., 2022), expanding the CRISPR/Cas9 system for epigenome manipulation (**Figure 1**). However, the first attempts at epigenomic editing with this system, which consisted of dCas9 fused with a single catalytic domain, did not result in effective editing, hence later work aimed at improving its efficiency (Pflueger et al., 2018). An ameliorated dCas9-derived strategy relies on additional proteins or domains interacting with the dCas9 system to recruit multiple copies of the effector. Such a case is used in dCas9- *Emesvirus zinderi*, commonly known as MS2 systems, where two loops in the sgRNA allow the recruitment of four MS2 bacteriophage coat proteins fused to effectors, increasing their modulating effects (Lee et al., 2019). The currently most used tool, however, is the SunTag system. SunTag is a protein-tagging system originally developed by Tanenbaum et al. (2014) that consists of 24 repeated General Control Nondepressible 4 (GCN4) moieties, originally linked by 5-aminoacid peptides and later optimized with 22-aminoacid linkers (Morita et al., 2023). First used for GFP-mediated protein detection, its structure allows to recruit multiple proteins fused with anti-GCN4 antibodies (scFv) on the GCN4 tail: the fusion of SunTag with dCas9 can therefore be used to recruit specific proteins in proximity to targeted DNA loci, directing their epigenetic activity towards selected genes. The SunTag-based system was also employed contextually to nucleases present in other CRISPR systems, most notably CRISPR from *Prevotella* and *Francisella*1 (Cpf1), a nuclease that naturally requires a single RNA guide and different PAMs in comparison to Cas9 (Swarts and Jinek, 2018). While the dCpf1 system has been used rarely up until now, the selection of an editing tool with different PAMs could both broaden the potential target sequences and reduce the number of off-targets.

Although CRISPR systems are abundantly used, recent work suggests a potential activity of Cas proteins in preventing other endogenous factors to properly access chromatin, resulting, for instance, in locally reduced epigenetic marks that do not directly depend on the active domains fused to the Cas protein (Oberkofler and Bäurle, 2022). Their remarkable versatility and multiplexing capability, however, makes them still the most prominently used editing tool.

The aim of this review is to describe all the currently available epigenome editing systems for plants epigenome. While more detailed reviews are available to compare these tools (see Wang and Yamaguchi, 2024) (Wang and Yamaguchi, 2024), this work aims to provide a brief overview of the results achieved this far in plants and the potential future direction for crop improvement.

## 2 Targeted epigenomic modifications in plants

Epigenetic mechanisms are extremely important for a proper control of transcription, especially in plants, which, as sessile organisms, must finely tune their responses with their surroundings, to reshape their responses accordingly. As for ZFs-based technology, only few reports have shown its application to edit plant organisms. However, with the advent of CRISPR/dCas systems, a general increase in plant editing and the possibility to perform epigenome editing was observed in *Arabidopsis thaliana* (hereafter Arabidopsis) as well as in some crop species, with the aim to target different epigenetic marks on a few model loci (See **Table 1**).

**Table 1:** Epigenetic modifications exploited in plants through epigenome editing tools.

Epigenetic modification	DNA methylation							DNA demethylation					Histone methylation	Histone acetylation		Histone demethylation	
**Targeted gene**	*FWA*						*OsGBSS1*	*FWA*		*CACTA1*		*FIE1*	*FT*	*FT*	*AREB1*	*CUC3*	*APX2*
**Expression effects**	Not investigated	DOWN	DOWN	DOWN	DOWN	DOWN	DOWN	UP	UP	UP	UP	UP	DOWN	UP (only T1 generation)	UP	UP	DOWN
**Editing tool**	ZF-SUVH2	dCas9-MQ1	dCas9-SunTag-MQ1	dCas9-SunTag-StkyC	ZF-SLN	dCas9-NtDRM	Cas12j2-DNMT3A/L	dCpf1-SunTag-TET1cd	ZF-TET1	dCpf1-SunTag-TET1cd	ZF-TET1	dCas9 -TET1	dCAS-MS2-KYP	dCAS9-MS2-p300	dCAS9-HAT1	dCas9-JMJC	dCas9-JMJ
**Plant model**	*A. thaliana*	*A. thaliana*	*A. thaliana*	*A. thaliana*	*A. thaliana*	*A. thaliana*	*O. sativa*	*A. thaliana*	*A. thaliana*	*A. thaliana*	*A. thaliana*	*O. sativa*	*A. thaliana*	*A. thaliana*	*A. thaliana*	*A. thaliana*	*A. thaliana*
**Phenotype**	Early flowering	Early flowering (after the T1 generation)	Early flowering	Early flowering	Not reported	Early flowering	Not observable	Not reported	Late flowering	Not observable	Not observable	Dwarf plant	Late flowering	Early flowering	Drought tolerance	Branching and short meristems	Not reported
**Reference**	Johnson et al. 2014	Ghoshal et al. 2021	Ghoshal et al. 2021	Boone et al. 2023	Ichino et al. 2021	Papikian et al. 2019	Liu et al. 2022	Zheng and He 2023	Gallego Bartolomé et al. 2018	Zheng and He 2023	Gallego Bartolomé et al. 2018	Tang et al. 2022	Lee et al. 2019	Lee et al. 2019	Roca Paixão et al., 2019	Fal et al. 2024	Oberkofler et al. 2022

### 2.1 DNA methylation

Among the first attempts at epigenome editing, it is worth mentioning the recruitment of the m5C reader SUVH2 on the *FLOWERING LOCUS WAGENINEN (FWA*) locus to dissect the mechanism of RdDM (Soppe et al., 2000; Wang et al., 2023). In plants, such recruitment of RNA Pol V to target loci is directed by SUVH2. To prove this interaction, Johnson and collaborators (Johnson et al., 2014) used a ZF-SUVH2 system to induce the methylation of *fwa-4* epiallele. *FWA* is a repressor of flowering transition, and the gene appears to be repressed due to its heavy methylation state in wild type plants. Conversely, the *fwa-4* epiallele is not methylated and a late flowering phenotype is displayed in Arabidopsis mutants (Soppe et al., 2000; Ikeda et al., 2007)

Through a ZF-SUVH2 system, the methylation state was restored in *fwa-4* mutants and transformed plants showed an acceleration in flowering time when compared to *fwa-4* single mutants. This methylation effect was maintained trans-generationally and bisulphite sequencing confirmed the methylation state of the locus for three generations. However, despite the DNA methylation events, the ZF-SUVH2 complex was not detected on *FWA* Chromatin immunoprecipitation, indicating a very dynamic process (Ikeda et al., 2007; Johnson et al., 2014). Alternatively, Ichino and collaborators (Ichino et al., 2021) targeted the *fwa-4* gene using a zinc finger (ZF) protein to evaluate the repressor activity of SILENZIO (SLN). SLN was identified through the study of two methyl CpG binding domain (MBD) proteins, MBD5 and MBD6. Plants transformed with ZF-SLN exhibited downregulation of *fwa-4*, even in the absence of methylation (Ichino et al., 2021). However, this approach also resulted in the downregulation of off-targets, highlighting the strong repressor activity of SLN but also the limited specificity of ZF.

A ZF-based system was also used to recruit human Ten Eleven Translocation (TET)1 DNA demethylase to *FWA* and *CACTA1* transposon (Gallego-Bartolomé et al., 2018). This resulted in an increased *FWA* expression and late flowering, a phenotype maintained across generations, even when the transgene was lost. Demethylation was also shown to be highly specific to the *FWA* locus without off-targets. In contrast, targeting the heavily methylated *CACTA1* transposon, located in heterochromatic genome regions, produced different results. Indeed, *CACTA1* demethylation was not sustained across generations in the absence of the transgene. While transgenic plants showed increased expression of *CACTA1*, this effect was lost after outcrossing the construct. Additionally, ZF-CACTA1-TET1 plants exhibited reduced global DNA methylation, highlighting differential methylation dynamics between loci. The study also employed a SunTag-dCas9 system to target the same loci. For *FWA*, results mirrored those obtained with the ZF system. However, for *CACTA1* demethylation occurred without the global effect observed with ZF, with genome-wide methylation levels resembling those of wild-type plant (Gallego-Bartolomé et al., 2018)

The promising results achieved using the ZF system have been applied to further investigate the RdDM pathway. By employing a combination of RdDM pathway mutants and an unmethylated *fwa-4* epiallele, Gallego-Bartolomé and collaborators (Gallego-Bartolomé et al., 2019) elucidated the hierarchical relationships among the various components of the pathway. A notable example of this work involves the use of Pol IV fused with ZF in *fwa-4* plants, which induced early flowering and methylation of the target locus. Furthermore, mutant plants for the histone reader SHH1 also showed methylation of the *fwa-4* epiallele when transformed with the ZF-Pol IV construct. Similarly, methylation of *fwa-4* was observed in different mutants for siRNAs production, *dcl2*, *dcl3*, and *dcl4*, when transformed with ZF-Pol IV. In contrast, methylation was not triggered in *rdr2* mutants, highlighting the essential role of RDR2 in this process. The results obtained with the ZF system prompted the use of another epigenome editing tool to modify the methylation state of the *FWA* allele. Thus, the dCas9-SunTag system on the Arabidopsis *fwa-4* mutant was targeted with the *Nicotiana tabacum* DNA methyltransferase enzyme (NtDRM) (Papikian et al., 2019). To induce the methylation of *fwa-4*, three different gRNAs were designed to recruit the system on the *FWA* locus. This led to the effective methylation in *fwa-4* seedlings and the phenotype was reverted as previously described. However, at least two generations containing dCas9-SunTag-NtDRM were required to induce a transgenerational effect for maintaining methylation on the targeted locus and early flowering phenotype. Despite the successful methylation of the target locus, many off-targets were detected as well as a general increase of DNA methylation at the whole genome level (Papikian et al., 2019). Even if this tool has been proven to be a powerful epigenome editor, off-target methylation should be further investigated to understand how to re-direct the methylation.

The *FWA* epiallele was targeted with greater specificity using a dCas9-SunTag system fused to the bacterial methyltransferase MQ1 from *Mollicutes* sp*iroplasma* (**Figure 1**) (Ghoshal et al., 2021). Arabidopsis plants transformed with the dCas9-SunTag-MQ1 system exhibited an early flowering phenotype as early as the T1 generation, which was accompanied by reduced *fwa-4* expression and increased methylation at the locus. Notably, these transformed plants showed a limited increase in genome-wide DNA methylation. The same phenotypic and molecular changes observed in the T1 generation persisted in the T2 generation and in progeny that had segregated away the transgene. In the same study, the *FWA* epiallele was also targeted using a dCas9-MQ1 system. Unlike the SunTag approach, T1 plants did not exhibit early flowering; however, a slight increase in DNA methylation at the target locus was observed, with no significant changes in genome-wide DNA (Ghoshal et al., 2021). Accordingly, T2 plants displayed increased DNA methylation levels at the *FWA* epiallele, which were associated with the induction of early flowering. Notably, these effects were sustained in plants even after the transgene was segregated out (Ghoshal et al., 2021). These findings confirmed the heritability of DNA methylation changes at the *FWA* locus and demonstrated the high specificity of the SunTag system, likely attributed to its ability to recruit a higher number of effectors to the target gene.

*FWA* was studied also to dissect the function of the previously mentioned MBD5/6 proteins (Boone et al., 2023). In particular, the StkyC domain of MBD6 was fused with a dCas9-SunTag system and recruited on the *fwa* unmethylated epiallele. Transformed plants displayed early flowering phenotype and downregulation of *fwa.* However, DNA methylation levels were not reported (Boone et al., 2023).

Epigenome editing has been applied not only to flowering genes and transposons but also to the gene encoding the enzyme granule-bound starch synthase 1 (GBSS1). In this case a Cas12j2 system, originally discovered in huge phages and with a size about half of the traditional Cas, was fused to the human DNA methyltransferase enzyme DNMT3A/L. The transformation of rice protoplasts with the system led to reduced gene expression by methylating *GBSS1* promoter region (Liu et al., 2022; Liu et al., 2024).

Similarly, the dCpf1-SunTag system was employed to demethylate the *FWA* locus and the *CACTA1* transposon by recruiting TET1 (Zheng and He, 2023). While the phenotype of the treated plants was not assessed, the methylation level of these loci was reported to decrease (Zheng and He, 2023), hinting at this system as an alternative and effective approach for targeted epigenetic manipulation.

The SunTag-dCas9-TET1 system was also applied in *Oryza sativa* to target the *FERTILIZATION-INDEPENDENT ENDOSPERM1* (*FIE1*) that induces a dwarf phenotype in rice when active (Tang et al., 2022). Indeed, a significant decrease of methylation was detected, correlated with an increase of *FIE1* mRNA, in line with the dwarf phenotype of the plants. A genome-wide DNA methylation analysis was performed in T2 plants, and a general lower methylation level was detected compared to the wild type. Overall, rice plants inherited a dwarf phenotype and de-methylation of *FIE1* in the absence of the transgene (Tang et al., 2022).

### 2.2 Histone methylation

Histone methylation was edited through a dCas9 system to recruit on *FLOWERING LOCUS T (FT)* the KRYPTONITE (KYP) enzyme in Arabidopsis. Generally, *ft* mutants show a late flowering phenotype while the over-expressing plants have an early flowering phenotype (Turck et al., 2008). Wild-type plants transformed with the construct dCas9-MS2-KYP displayed a late flowering phenotype in the T1 generation, in line with the expected effects of H3K9 di-methylation on gene expression. Interestingly, the same phenotype of the T1 was displayed in the T2. Surprisingly, H3K9 methylation on the *FT* locus was not detected in T3. Flowering time was not assessed in T3 (Lee et al., 2019).

Targeted removal of H3K4 methylation was performed through a dCas9-based system in fusion with the catalytic domain of JMJ18 to target the heat stress-responsive *ASCORBATE PEROXIDASE 2* (*APX2*) gene (Oberkofler and Bäurle, 2022). This locus was observed to be primed in response to heat stress, showing an enhanced H3K4me3 enrichment upon a second exposure to high temperatures (Oberkofler and Bäurle, 2022). The employment of dCas9-JMJ18, however, resulted in an overall decrease in H3K4me3 suggesting epigenome editing as a tool to alter priming stress mechanisms (Oberkofler and Bäurle, 2022).

An additional application of dCas9-based tools to promote targeted demethylation relied on the C-terminal (JMJC) domain from JMJ13, an Arabidopsis H3K27me3 demethylase. This dCas9-JMJC system was used by Fal and collaborators to evict H3K27me3 from *CUP SHAPED COTYLEDONS 3* (*CUC3*), a gene involved in shoot apical meristem initiation and maintenance. Lines with hypomethylated *CUC3* showed an enhanced and ectopic expression of the gene. This change was reflected by the observed phenotype, as mutant plants showed multiple stems arising from rosettes and shorter meristems (Fal et al., 2024).

### 2.3 Histone acetylation

The dCas9-MS2-effector system was employed to recruit the human acetyltransferase p300 to the *FT* locus in Arabidopsis. In the T1 generation transformed with the MS2-p300 construct, plants exhibited an early flowering phenotype, consistent with gene expression changes associated with H3K27 acetylation (Lee et al., 2019). However, in the T2 generation, this phenotype was not observed, suggesting possible epigenetic resetting or instability across generations. Although *FT* expression levels were comparable to those of wild-type plants, an increase in H3K27 acetylation was still observed in generation T3 (Lee et al., 2019). Further investigations are requested to determine possible counteracting effects to the deposition of histone acetylation.

In another study, the *ABA-responsive element binding protein 1 (AREB1/ABF2)*, encoding a critical regulator of drought stress response, was targeted by fusing directly dCas9 with the Histone Acetyl Transferase 1 (HAT1) (Roca Paixão et al., 2019). Notably, dCas9-HAT1-transformed plants showed enhanced survival under both mild and severe drought stress conditions compared to wild-type plants. This transformation also led to increased *AREB1* expression, although histone acetylation at the *AREB1* locus was not investigated, and, therefore, indirect effects cannot be excluded (Roca Paixão et al., 2019).

## 3 Conclusions and future perspectives

With the increasing number of tools developed in gene editing technologies, it will soon be possible to generate and introduce novel epi-alleles in innovative crop-breeding programs. This also comprises spontaneous epimutations identified in crop wild relatives that can be considered as a new source of variability with additional value to phenotypic diversity, including improving tolerance to abiotic and biotic stresses. However, the biology underpinning plant development and growth is highly variable, therefore it remains complex to address the relationships between the effect mediated by the epigenome tools and the putative ameliorated features.

Whilst these tools can be used to increase our understanding of the function of a specific epigenetic modification (e.g. DNA methylation, histone methylation or acetylation) over a defined set of genes, there are several challenges that still need to be addressed. Among them, reducing the number of off-targets effects and improving transgenerational stability are imperative. Thus, the design of novel dCas variants (e.g. enhanced dCas12), synthetically reintroduced histone variants and the expansion of guide RNA design tools represent promising strategies to further improve the system. Nevertheless, it remains important to consider the impact that biotic and abiotic stress could have on the epigenetic landscape and how they can affect the outcome of epigenome editing. Indeed, they could generate a priming effect that could be carried on by the offspring. Together with that, expanding the epi-tools with the implementation of other histone modifications such as ubiquitination or phosphorylation, will add more depth into mechanisms such as DNA repair and increase the chances to generate novel recombination hotspots. Furthermore, the evidence that some of these changes behave in a transgenerational manner opens to the possibility to accelerate breeding programs and to bypass genetic transformation.

To achieve these tasks, multidisciplinary strategies are required, involving not only geneticists and breeders, but also experts in mathematics, synthetic biology and biotechnology. Indeed, combining epigenome editing with computational tools and advanced delivery systems or nucleosome turnover already developed and assessed in bacteria and mammals will further enhance the generation of plant epialleles. Additionally, a comprehensive approach will also allow to clarify ethical concerns regarding epigenome editing, by offering solutions such as traceability and unambiguous risk and safety assessments. Altogether a cohesive and integrative work remains the key to harness the full potential behind the new generation of gene editing in plants.

## References

[B1] Barrangou R. Fremaux C. Deveau H. Richards M. Boyaval P. Moineau S.. (2007). CRISPR provides acquired resistance against viruses in prokaryotes. Sci. (1979) 315, 1709–1712. doi: 10.1126/science.1138140 17379808

[B2] Beerli R. R. Barbas C. F. (2002). Engineering polydactyl zinc-finger transcription factors. Nat. Biotechnol. 20, 135–141. Available at: https://www.nature.com/articles/nbt0202-135. (Accessed December 05, 2024) 11821858 10.1038/nbt0202-135

[B3] Boch J. Bonas U. (2010). Xanthomonas AvrBs3 family-type III effectors: Discovery and function. Annu. Rev. Phytopathol. 48, 419–436. doi: 10.1146/annurev-phyto-080508-081936 19400638

[B4] Boone B. A. Ichino L. Wang S. Gardiner J. Yun J. Jami-Alahmadi Y.. (2023). ACD15, ACD21, and SLN regulate the accumulation and mobility of MBD6 to silence genes and transposable elements. Sci. Adv. 9, 1–14. doi: 10.1126/sciadv.adi9036 PMC10651127 37967186

[B5] Candela-Ferre J. Diego-Martin B. Pérez-Alemany J. Gallego-Bartolomé J. (2024). Mind the gap: Epigenetic regulation of chromatin accessibility in plants. Plant Physiol. 194, 1998–2016. doi: 10.1093/plphys/kiae024 38236303 PMC10980423

[B6] Cano-Rodriguez D. Rots M. G. (2016). Epigenetic editing: on the verge of reprogramming gene expression at will. Curr. Genet. Med. Rep. 4, 170–179. doi: 10.1007/s40142-016-0104-3 27933223 PMC5119838

[B7] Cedar H. Bergman Y. (2009). Linking DNA methylation and histone modification: patterns and paradigms. Nat. Rev. Genet. 10, 295–304. Available at: https://www.nature.com/articles/nrg2540. (Accessed December 10, 2024) 19308066 10.1038/nrg2540

[B8] Cermak T. Doyle E. L. Christian M. Wang L. Zhang Y. Schmidt C.. (2011). Efficient design and assembly of custom TALEN and other TAL effector-based constructs for DNA targeting. Nucleic Acids Res. 39, e82–e82. doi: 10.1093/nar/gkr218 21493687 PMC3130291

[B9] Charpentier E. Richter H. van der Oost J. White M. F. (2015). Biogenesis pathways of RNA guides in archaeal and bacterial CRISPR-Cas adaptive immunity. FEMS Microbiol. Rev. 39, 428–441. doi: 10.1093/femsre/fuv023 25994611 PMC5965381

[B10] Chen H. Kazemier H. G. De Groote M. L. Ruiters M. H. J. Xu G. L. Rots M. G. (2014). Induced DNA demethylation by targeting Ten-Eleven Translocation 2 to the human ICAM-1 promoter. Nucleic Acids Res. 42, 1563–1574. doi: 10.1093/nar/gkt1019 24194590 PMC3919596

[B11] Crevillén P. (2020). Histone demethylases as counterbalance to H3K27me3 silencing in plants. iScience 23. Available at: http://www.cell.com/article/S2589004220309123/fulltext. 10.1016/j.isci.2020.101715 PMC7649346 33205025

[B12] Duan C. Zhang H. Tang K. Zhu X. Qian W. Hou Y.. (2015). Specific but interdependent functions for A rabidopsis AGO 4 and AGO 6 in RNA -directed DNA methylation. EMBO J. 34, 581–592. doi: 10.15252/embj.201489453 25527293 PMC4365029

[B13] Fal K. Le Masson M. Berr A. Carles C. C. (2024). Manipulating plant development by editing histone methylation with the dCas9 tool: the CUC3 boundary gene as a case study. bioRxiv. 2873, 302–332 doi: 10.1101/2024.03.18.585636v1

[B14] Gallego-Bartolomé J. Gardiner J. Liu W. Papikian A. Ghoshal B. Kuo H. Y.. (2018). Targeted DNA demethylation of the arabidopsis genome using the human TET1 catalytic domain. Proc. Natl. Acad. Sci. U.S.A. 115, E2125–E2134. Available at: https://pmc.ncbi.nlm.nih.gov/articles/PMC5834696/. 29444862 10.1073/pnas.1716945115 PMC5834696

[B15] Gallego-Bartolomé J. Liu W. Kuo P. H. Feng S. Ghoshal B. Gardiner J.. (2019). Co-targeting RNA polymerases IV and V promotes efficient de novo DNA methylation in arabidopsis. Cell 176, 1068. Available at: https://pmc.ncbi.nlm.nih.gov/articles/PMC6386582/. 30739798 10.1016/j.cell.2019.01.029 PMC6386582

[B16] Gao X. Tsang J. C. H. Gaba F. Wu D. Lu L. Liu P. (2014). Comparison of TALE designer transcription factors and the CRISPR/dCas9 in regulation of gene expression by targeting enhancers. Nucleic Acids Res. 42, e155–e155. doi: 10.1093/nar/gku836 25223790 PMC4227760

[B17] Garneau J. E. Dupuis M. È. Villion M. Romero D. A. Barrangou R. Boyaval P.. (2010). The CRISPR/Cas bacterial immune system cleaves bacteriophage and plasmid DNA. Nature 468, 67–71. Available at. doi: 10.1038/nature09523 21048762

[B18] Ghoshal B. Picard C. L. Vong B. Feng S. Jacobsen S. E. (2021). CRISPR-based targeting of DNA methylation in Arabidopsis thaliana by a bacterial CG-specific DNA methyltransferase. Proc. Natl. Acad. Sci. U.S.A. 118, e2125016118. doi: 10.1073/pnas.2125016118 34074795 PMC8201958

[B19] Grimmer M. R. Stolzenburg S. Ford E. Lister R. Blancafort P. Farnham P. J. (2014). Analysis of an artificial zinc finger epigenetic modulator: widespread binding but limited regulation. Nucleic Acids Res. 42, 10856–10868. doi: 10.1093/nar/gku708 25122745 PMC4176344

[B20] Guo J. Gaj T. Barbas C. F. (2010). Directed evolution of an enhanced and highly efficient FokI cleavage domain for zinc finger nucleases. J. Mol. Biol. 400, 96–107. Available at. doi: 10.1016/j.jmb.2010.04.060 20447404 PMC2885538

[B21] Haag J. R. Ream T. S. Marasco M. Nicora C. D. Norbeck A. D. Pasa-Tolic L.. (2012). *In vitro* transcription activities of pol IV, pol V, and RDR2 reveal coupling of pol IV and RDR2 for dsRNA synthesis in plant RNA silencing. Mol. Cell. 48, 811–818. doi: 10.1016/j.molcel.2012.09.027 23142082 PMC3532817

[B22] Henderson I. R. Zhang X. Lu C. Johnson L. Meyers B. C. Green P. J.. (2006). Dissecting Arabidopsis thaliana DICER function in small RNA processing, gene silencing and DNA methylation patterning. Nat. Genet. 38, 721–725. Available at: https://www.nature.com/articles/ng1804. (Accessed January 13, 2025) 16699516 10.1038/ng1804

[B23] Hensel G. Kumlehn J. (2019). Genome engineering using TALENs. Methods Mol. Biol. 1900, 195–215. doi: 10.1007/978-1-4939-8944-7_13 30460567

[B24] Hsu P. D. Lander E. S. Zhang F. (2014). Development and applications of CRISPR-Cas9 for genome engineering. Cell 157, 1262–1278. Available at. doi: 10.1016/j.cell.2014.05.010 24906146 PMC4343198

[B25] Ichino L. Boone B. A. Strauskulage L. Harris C. J. Kaur G. Gladstone M. A.. (2021). MBD5 and MBD6 couple DNA methylation to gene silencing through the J-domain protein SILENZIO. Sci. (1979) 372, 1434–1439. doi: 10.1126/science.abg6130 PMC8639832 34083448

[B26] Ikeda Y. Kobayashi Y. Yamaguchi A. Abe M. Araki T. (2007). Molecular basis of late-flowering phenotype caused by dominant epi-alleles of the FWA locus in arabidopsis. Plant Cell Physiol. 48, 205–220. doi: 10.1093/pcp/pcl061 17189287

[B27] Jankele R. Svoboda P. (2014). TAL effectors: tools for DNA Targeting. Brief Funct. Genomics 13, 409–419. doi: 10.1093/bfgp/elu013 24907364 PMC4168661

[B28] Jiang W. Bikard D. Cox D. Zhang F. Marraffini L. A. (2013). RNA-guided editing of bacterial genomes using CRISPR-Cas systems. Nat. Biotechnol. 31, 233–239. Available at: https://www.nature.com/articles/nbt.2508. (Accessed January 13, 2025) 23360965 10.1038/nbt.2508 PMC3748948

[B29] Johnson L. M. Du J. Hale C. J. Bischof S. Feng S. Chodavarapu R. K.. (2014). SRA- and SET-domain-containing proteins link RNA polymerase V occupancy to DNA methylation. Nature 507, 124–128. Available at: https://www.nature.com/articles/nature12931. (Accessed October 29, 2024) 24463519 10.1038/nature12931 PMC3963826

[B30] Kharchenko P. V. Alekseyenko A. A. Schwartz Y. B. Minoda A. Riddle N. C. Ernst J.. (2010). Comprehensive analysis of the chromatin landscape in Drosophila melanogaster. Nature 471, 480–485. Available at: https://www.nature.com/articles/nature09725. (Accessed December 05, 2024) 21179089 10.1038/nature09725 PMC3109908

[B31] Kim S. Y. Lee J. Eshed-Williams L. Zilberman D. Sung Z. R. (2012). EMF1 and PRC2 cooperate to repress key regulators of arabidopsis development. PLoS Genet. 8, e1002512. doi: 10.1371/journal.pgen.1002512 22457632 PMC3310727

[B32] Lauria M. Rossi V. (2011). Epigenetic control of gene regulation in plants. Biochim. Biophys. Acta (BBA) - Gene Regul. Mechanisms. 1809, 369–378. doi: 0.1016/j.bbagrm.2011.03.002 10.1016/j.bbagrm.2011.03.002 21414429

[B33] Law J. A. Du J. Hale C. J. Feng S. Krajewski K. Palanca A. M. S.. (2013). Polymerase IV occupancy at RNA-directed DNA methylation sites requires SHH1. Nature 498, 385–389. Available at: https://www.nature.com/articles/nature12178. (Accessed January 09, 2025) 23636332 10.1038/nature12178 PMC4119789

[B34] Lee J. E. Neumann Id M. Duro D. I. Schmid Id M. (2019). CRISPR-based tools for targeted transcriptional and epigenetic regulation in plants. PLoS ONE 14 (9), 1–17. doi: 10.1371/journal.pone.0222778 PMC6762090 31557222

[B35] León-Ruiz J. Espinal-Centeno A. Blilou I. Scheres B. Arteaga-Vázquez M. Cruz-Ramírez A. (2023). RETINOBLASTOMA-RELATED interactions with key factors of the RNA-directed DNA methylation (RdDM) pathway and its influence on root development. Planta 257, 1–11. doi: 10.1007/s00425-023-04135-x 37120771

[B36] Liu S. Sretenovic S. Fan T. Cheng Y. Li G. Qi A.. (2022). Hypercompact CRISPR–Cas12j2 (CasΦ) enables genome editing, gene activation, and epigenome editing in plants. Plant Commun. 3, 100453. Available at: https://pmc.ncbi.nlm.nih.gov/articles/PMC9700201/. 36127876 10.1016/j.xplc.2022.100453 PMC9700201

[B37] Liu S. Tang X. Qi Y. Zhang Y. (2024). “Optimizing rice genomics: employing the hypercompact cas12j2 system for targeted transcriptional regulation and epigenome modification,” in Synthetic Promoters: Methods and Protocols. Ed. Marchisio M. A. (Springer US, New York, NY), 133–143. doi: 10.1007/978-1-0716-4063-0_9 39068337

[B38] Loffer A. Singh J. Fukudome A. Mishra V. Wang F. Pikaard C. S. (2022). A DCL3 dicing code within Pol IV-RDR2 transcripts diversifies the siRNA pool guiding RNA-directed DNA methylation. Elife 11, 1–22. doi: 10.7554/eLife.73260 PMC8846587 35098919

[B39] Luger K. Mäder A. W. Richmond R. K. Sargent D. F. Richmond T. J. (1997). Crystal structure of the nucleosome core particle at 2.8 Å resolution. Nature 389, 251–260. Available at: https://www.nature.com/articles/38444. (Accessed December 04, 2024) 9305837 10.1038/38444

[B40] Mali P. Yang L. Esvelt K. M. Aach J. Guell M. DiCarlo J. E.. (2013). RNA-guided human genome engineering via Cas9. Sci. (1979) 339, 823–826. doi: 10.1126/science.1232033 PMC3712628 23287722

[B41] Mansisidor A. R. Risca V. I. (2022). Chromatin accessibility: methods, mechanisms, and biological insights. Nucleus 13, 236–276. doi: 10.1080/19491034.2022.2143106 36404679 PMC9683059

[B42] Miller J. McLachlan A. D. Klug A. (1985). Repetitive zinc-binding domains in the protein transcription factor IIIA from Xenopus oocytes. EMBO J. 4, 1609–1614. doi: 10.1002/j.1460-2075.1985.tb03825.x 4040853 PMC554390

[B43] Morita S. Horii T. Hatada I. (2023). Regulation of gene expression using dCas9-sunTag platforms. Methods Mol. Biol. 2577, 189–195. doi: 10.1007/978-1-0716-2724-2_13 36173574

[B44] Oberkofler V. Bäurle I. (2022). Inducible epigenome editing probes for the role of histone H3K4 methylation in Arabidopsis heat stress memory. Plant Physiol. 189, 703–714. doi: 10.1093/plphys/kiac113 35285498 PMC9157090

[B45] Onodera Y. Haag J. R. Ream T. Nunes P. C. Pontes O. Pikaard C. S. (2005). Plant nuclear RNA polymerase IV mediates siRNA and DNA methylation-dependent heterochromatin formation. Cell. 120, 613–622. doi: 10.1016/j.cell.2005.02.007 15766525

[B46] Papikian A. Liu W. Gallego-Bartolomé J. Jacobsen S. E. (2019). Site-specific manipulation of Arabidopsis loci using CRISPR-Cas9 SunTag systems. Nat. Commun. 10, 1–11. doi: 10.1038/s41467-019-08736-7 30760722 PMC6374409

[B47] Pflueger C. Tan D. Swain T. Nguyen T. Pflueger J. Nefzger C.. (2018). A modular dCas9-SunTag DNMT3A epigenome editing system overcomes pervasive off-target activity of direct fusion dCas9-DNMT3A constructs. Genome Res. 28, 1193–1206. Available at: https://genome.cshlp.org/content/28/8/1193.full. (Accessed October 24, 2024) 29907613 10.1101/gr.233049.117 PMC6071642

[B48] Qi L. S. Larson M. H. Gilbert L. A. Doudna J. A. Weissman J. S. Arkin A. P.. (2013). Repurposing CRISPR as an RNA-γuided platform for sequence-specific control of gene expression. Cell 152, 1173–1183. Available at: http://www.cell.com/article/S0092867413002110/fulltext. (Accessed December 05, 2024) 23452860 10.1016/j.cell.2013.02.022 PMC3664290

[B49] Roca Paixão J. F. Gillet F. X. Ribeiro T. P. Bournaud C. Lourenço-Tessutti I. T. Noriega D. D.. (2019). Improved drought stress tolerance in Arabidopsis by CRISPR/dCas9 fusion with a Histone AcetylTransferase. Sci. Rep. 9, 1–9. Available at: https://www-nature-com.pros2.lib.unimi.it/articles/s41598-019-44571-y. (Accessed November 06, 2024) 31147630 10.1038/s41598-019-44571-y PMC6542788

[B50] Saifaldeen M. Al-Ansari D. E. Ramotar D. Aouida M. (2020). CRISPR fokI dead cas9 system: principles and applications in genome engineering. Cells 9, 2518. Available at: https://www.mdpi.com/2073-4409/9/11/2518/htm. (Accessed December 05, 2024) 33233344 10.3390/cells9112518 PMC7700487

[B51] Sapranauskas R. Gasiunas G. Fremaux C. Barrangou R. Horvath P. Siksnys V. (2011). The Streptococcus thermophilus CRISPR/Cas system provides immunity in Escherichia coli. Nucleic Acids Res. 39, 9275–9282. doi: 10.1093/nar/gkr606 21813460 PMC3241640

[B52] Schmitz R. J. Lewis Z. A. Goll M. G. (2019). DNA Methylation: Shared and Divergent Features across Eukaryotes. Trends Genet. 35, 818–827. Available at: http://www.cell.com/article/S0168952519301453/fulltext. (Accessed December 10, 2024) 31399242 10.1016/j.tig.2019.07.007 PMC6825889

[B53] Schubert V. Rudnik R. Schubert I. (2014). Chromatin associations in Arabidopsis interphase nuclei. Front. Genet. 5, 107489. doi: 10.3389/fgene.2014.00389 PMC4230181 25431580

[B54] Segal D. J. Beerli R. R. Blancafort P. Dreier B. Effertz K. Huber A.. (2003). Evaluation of a modular strategy for the construction of novel polydactyl zinc finger DNA-binding proteins. Biochemistry 42, 2137–2148. Available at. doi: 10.1021/bi026806o 12590603

[B55] Sequeira-Mendes J. Aragüez I. Peiró R. Mendez-Giraldez R. Zhang X. Jacobsen S. E.. (2014). The functional topography of the arabidopsis genome is organized in a reduced number of linear motifs of chromatin states. Plant Cell 26, 2351–2366. Available at. doi: 10.1105/tpc.114.124578 24934173 PMC4114938

[B56] Shen Y. Wei W. Zhou D. X. (2015). Histone acetylation enzymes coordinate metabolism and gene expression. Trends Plant Sci. 20, 614–621. Available at: http://www.cell.com/article/S136013851500196X/fulltext. (Accessed December 04, 2024) 26440431 10.1016/j.tplants.2015.07.005

[B57] Siddique A. N. Nunna S. Rajavelu A. Zhang Y. Jurkowska R. Z. Reinhardt R.. (2013). Targeted methylation and gene silencing of VEGF-A in human cells by using a designed dnmt3a–dnmt3L single-chain fusion protein with increased DNA methylation activity. J. Mol. Biol. 425, 479–491. doi: 10.1016/j.jmb.2012.11.038 23220192

[B58] Soppe W. J. J. Jacobsen S. E. Alonso-Blanco C. Jackson J. P. Kakutani T. Koornneef M.. (2000). The late flowering phenotype of fwa mutants is caused by gain-of-function epigenetic alleles of a homeodomain gene. Mol. Cell. 6, 791–802. doi: 10.1016/S1097-2765(05)00090-0 11090618

[B59] Swarts D. C. Jinek M. (2018). Cas9 versus Cas12a/Cpf1: Structure–function comparisons and implications for genome editing. Wiley Interdiscip Rev. RNA 9, e1481. doi: 10.1002/wrna.1481 29790280

[B60] Tanenbaum M. E. Gilbert L. A. Qi L. S. Weissman J. S. Vale R. D. (2014). A protein-tagging system for signal amplification in gene expression and fluorescence imaging. Cell 159, 635–646. Available at. doi: 10.1016/j.cell.2014.09.039 25307933 PMC4252608

[B61] Tang S. Yang C. Wang D. Deng X. Cao X. Song X. (2022). Targeted DNA demethylation produces heritable epialleles in rice. Sci. China Life Sci. 65, 753–756. doi: 10.1007/s11427-021-1974-7 34406573

[B62] Turck F. Fornara F. Coupland G. (2008). Regulation and identity of florigen: Flowering Locus T moves center stage. Annu. Rev. Plant Biol. 59, 573–594. doi: 10.1146/annurev.arplant.59.032607.092755 18444908

[B63] Wang M. He L. Chen B. Wang Y. Wang L. Zhou W.. (2022). Transgenerationally transmitted DNA demethylation of a spontaneous epialleles using CRISPR/dCas9-TET1cd targeted epigenetic editing in arabidopsis. Int. J. Mol. Sci. 23, 10492. Available at: https://www.mdpi.com/1422-0067/23/18/10492/htm. (Accessed December 05, 2024) 36142407 10.3390/ijms231810492 PMC9504898

[B64] Wang X. Yamaguchi N. (2024). Cause or effect: Probing the roles of epigenetics in plant development and environmental responses. Curr. Opin. Plant Biol. 81, 102569. doi: 10.1016/j.pbi.2024.102569 38833828

[B65] Wang M. Zhong Z. Gallego-Bartolomé J. Li Z. Feng S. Kuo H. Y.. (2023). A gene silencing screen uncovers diverse tools for targeted gene repression in Arabidopsis. Nat. Plants. 9, 460–472. doi: 10.1038/s41477-023-01362-8 36879017 PMC10027610

[B66] Zhang H. Lang Z. Zhu J. K. (2018). Dynamics and function of DNA methylation in plants. Nat. Rev. Mol. Cell Biol. 19, 489–506. Available at: https://www.nature.com/articles/s41580-018-0016-z. (Accessed December 05, 2024) 29784956 10.1038/s41580-018-0016-z

[B67] Zheng Y. He R. (2023). Targeted DNA demethylation of the Arabidopsis genome using the SunTag-dCpf1-TET1cd system. MicroPubl Biol. doi: 0.17912/micropub.biology.000814 10.17912/micropub.biology.000814 PMC10074173 37033707

